# Supplementary material for: Parental Perception of the Oral Health-Related Quality of Life of Children and Adolescents with Autism Spectrum Disorder (ASD)
Source: Int J Environ Res Public Health. 2023 Jan 9;20(2):1151. doi: 10.3390/ijerph20021151 (PMC9859466; doi:10.3390/ijerph20021151)
Supplement: Supplementary file 1 [file ijerph-20-01151-s001.zip › ijerph-2082582-supplementary.pdf]

Supplementary Material

This supplementary material contains:

(1) the short versions of Brazilian Portuguese P-CPQ and FIS (16-P-CPQ and 4-FIS), developed from the most impactful items from the original questionnaires, translated and validate for Brazilian Portuguese language.
(2) the English version of the questions used in 16-P-CPQ and 4-FIS selected from the original questionnaires.

(1) Questionário de Saúde Bucal Infantil
Percepção dos pais (6–14 anos)
VERSÃO CURTA—16 itens

INSTRUÇÕES AOS PAIS

1. Esse questionário é sobre os efeitos das condições bucais no bem-estar e vida diária das crianças e desses efeitos sobre suas famílias durante a pandemia. Nós estamos interessados em qualquer condição que envolva dentes, lábios, boca e maxilares. **Por favor, responda cada questão**.
2. Para responder a questão, favor colocar um **X na caixa próxima à resposta.**
3. Por favor, dê a resposta que **melhor descrever a experiência de seu filho(a).** Se a questão não estiver de acordo com seu filho(a), favor responder "Nunca".
Exemplo: Com que frequência seu filho(a) teve dificuldade para prestar atenção na escola ou nas atividades online?
Se seu filho(a) teve dificuldade para prestar atenção na escola ou nas atividades online devido a problemas com os dentes, lábios, boca ou maxilares, escolha a resposta apropriada. Se aconteceu por outras razões, escolha "Nunca".
[ ] Nunca [ ] Uma ou duas vezes [ ] Algumas vezes [ ] Frequentemente [ ] Todos os dias ou quase todos os dias [ ] Não sei

Por favor, não discuta as questões com seu filho(a), pois estamos apenas interessados na opinião dos pais nesse questionário.

SEÇÃO 1: Saúde bucal e bem-estar da criança

1. Como você classificaria a saúde dos dentes, lábios, maxilares e boca de seu filho(a)?
[ ] Excelente [ ] Muito boa [ ] Boa [ ] Regular [ ] Ruim

2. Quanto o bem-estar geral de seu filho(a) é afetado pela condição de seus dentes, lábios, maxilares ou boca?
[ ] Nenhum pouco [ ] Muito pouco [ ] Um pouco [ ] Muito [ ] Bastante

SEÇÃO 2: As questões a seguir são sobre **sintomas e desconfortos** que as crianças podem sentir devido às **condições de seus dentes, lábios, boca e maxilares**

SINTOMAS ORAIS

Durante **os últimos 3 meses**, com que frequência:

3. Seu filho(a) teve dor nos dentes, lábios, maxilares ou boca?
[ ] Nunca [ ] Uma ou duas vezes [ ] Algumas vezes [ ] Frequentemente [ ] Todos os dias ou quase todos os dias [ ] Não sei

5. Seu filho(a) teve machucados na boca?
[ ] Nunca [ ] Uma ou duas vezes [ ] Algumas vezes [ ] Frequentemente [ ] Todos os dias ou quase todos os dias [ ] Não sei

6. Seu filho(a) teve mau hálito?

[ ] Nunca [ ] Uma ou duas vezes [ ] Algumas vezes [ ] Frequentemente [ ] Todos os dias ou quase todos os dias [ ] Não sei

8. Seu filho(a) teve alimento preso dentro ou entre os dentes?
[ ] Nunca [ ] Uma ou duas vezes [ ] Algumas vezes [ ] Frequentemente [ ] Todos os dias ou quase todos os dias [ ] Não sei

LIMITAÇÕES FUNCIONAIS

9. Seu filho(a) teve dificuldade de morder ou mastigar comidas como maçã, espiga de milho ou carne dura?
[ ] Nunca [ ] Uma ou duas vezes [ ] Algumas vezes [ ] Frequentemente [ ] Todos os dias ou quase todos os dias [ ] Não sei

10. Seu filho(a) respirou pela boca?
[ ] Nunca [ ] Uma ou duas vezes [ ] Algumas vezes [ ] Frequentemente [ ] Todos os dias ou quase todos os dias [ ] Não sei

11. Seu filho(a) teve problemas durante o sono?
[ ] Nunca [ ] Uma ou duas vezes [ ] Algumas vezes [ ] Frequentemente [ ] Todos os dias ou quase todos os dias [ ] Não sei

14. Seu filho(a) teve dificuldade para beber ou comer alimentos quentes ou frios?
[ ] Nunca [ ] Uma ou duas vezes [ ] Algumas vezes [ ] Frequentemente [ ] Todos os dias ou quase todos os dias [ ] Não sei

SEÇÃO 3: As questões a seguir perguntam sobre os efeitos que **a condição dos dentes, lábios, boca e maxilares de seu filho(a)** podem ter no **sentimento** e **nas atividades diárias deles.**

BEM-ESTAR EMOCIONAL

Durante **os últimos 3 meses**, devido aos **dentes, lábios, boca ou maxilares**, com que frequência:

18. Seu filho(a) se sente irritado(a) ou frustrado(a)?
[ ] Nunca [ ] Uma ou duas vezes [ ] Algumas vezes [ ] Frequentemente [ ] Todos os dias ou quase todos os dias [ ] Não sei

19. Seu filho(a) se sente ansioso ou com medo?
[ ] Nunca [ ] Uma ou duas vezes [ ] Algumas vezes [ ] Frequentemente [ ] Todos os dias ou quase todos os dias [ ] Não sei

28. Seu filho (a) agiu timidamente ou com vergonha?
[ ] Nunca [ ] Uma ou duas vezes [ ] Algumas vezes [ ] Frequentemente [ ] Todos os dias ou quase todos os dias [ ] Não sei

34. Seu filho(a) se sentiu preocupado(a) com o que outras pessoas pensam sobre os dentes, lábios, boca ou maxilares?
[ ] Nunca [ ] Uma ou duas vezes [ ] Algumas vezes [ ] Frequentemente [ ] Todos os dias ou quase todos os dias [ ] Não sei

BEM-ESTAR SOCIAL

Durante **os últimos 3 meses**, devido aos **dentes, lábios, boca ou maxilares**, com que frequência:

20. Seu filho(a) faltou à escola ou deixou de participar das atividades online (ex. dor, consultas, cirurgias)?
[ ] Nunca [ ] Uma ou duas vezes [ ] Algumas vezes [ ] Frequentemente [ ] Todos os dias ou quase todos os dias [ ] Não sei

21. Seu filho(a) teve dificuldade para prestar atenção na escola ou nas atividades online?
[ ] Nunca [ ] Uma ou duas vezes [ ] Algumas vezes [ ] Frequentemente [ ] Todos os dias ou quase todos os dias [ ] Não sei

24. Seu filho(a) evitou sorrir ou rir quando estava perto de outras crianças?
[ ] Nunca [ ] Uma ou duas vezes [ ] Algumas vezes [ ] Frequentemente [ ] Todos os dias ou quase todos os dias [ ] Não sei

29. Seu filho(a) foi provocado(a) ou apelidado(a) por outras crianças?
[ ] Nunca [ ] Uma ou duas vezes [ ] Algumas vezes [ ] Frequentemente [ ] Todos os dias ou quase todos os dias [ ] Não sei

4-FIS versão curta

SEÇÃO 4: As questões seguintes perguntam sobre efeitos que **a condição bucal de seu filho(a)** pode ter nos **PAIS OU OUTROS MEMBROS FAMILIARES**

ESCALA DE IMPACTO FAMILIAR (FIS)

Durante **os últimos 3 meses**, devido aos **dentes, lábio, boca ou maxilares de seu filho(a)**, com que frequência:

36. Você ou outro membro da família se sentiu perturbado?
[ ] Nunca [ ] Uma ou duas vezes [ ] Algumas vezes [ ] Frequentemente [ ] Todos os dias ou quase todos os dias [ ] Não sei

37. Você ou outro membro da família teve o sono interrompido?
[ ] Nunca [ ] Uma ou duas vezes [ ] Algumas vezes [ ] Frequentemente [ ] Todos os dias ou quase todos os dias [ ] Não sei

40. Você ou outro membro da família teve menos tempo para si mesmo ou para família?
[ ] Nunca [ ] Uma ou duas vezes [ ] Algumas vezes [ ] Frequentemente [ ] Todos os dias ou quase todos os dias [ ] Não sei

43. Seu filho(a) ficou com ciúmes de você ou de outros membros da família?
[ ] Nunca [ ] Uma ou duas vezes [ ] Algumas vezes [ ] Frequentemente [ ] Todos os dias ou quase todos os dias [ ] Não sei

---

A numeração das questões corresponde à numeração do questionário original [14-16].

(2) Parental-Caregiver Perception Questionnaire (P-CPQ) and Family Impact Scale (FIS)
(6-14 years)
SHORT FORM—16 items

INSTRUCTIONS TO PARENTS

1. This questionnaire is about the effects of oral conditions on children's well-being and everyday life, and the effects on their families. We are interested in any condition that involves teeth, lips, mouth or jaws. **Please answer each question.**
2. To answer the question please put an **X in the box by the response.**
3. Please give the response that **best describes your child's experience**. If the question does not apply to your child, please answer with "Never".
Example: How often has your child had a hard time paying attention in school?

If your child has had a hard time paying attention in school because of problems with his/her teeth, lips mouth or jaws, choose the appropriate response. If it has happened for other reasons, choose "Never".
[ ] Never [ ] Once or twice [ ] Sometimes [ ] Often [ ] Everyday or almost everyday [ ] Don't know

Please do not discuss the questions with your child, as we are interested only in the parents' perspective in this questionnaire.

SECTION 1: Child's oral health and well-being

1. How would you rate the health of your child's teeth, lips, jaws and mouth?
[ ] Excellent [ ] Very good [ ] Good [ ] Fair [ ] Poor

2. How much is your child's overall well-being affected by the condition of his/her teeth, lips, jaws or mouth?
[ ] Not at all [ ] A little [ ] Somewhat [ ] A lot [ ] Very much

SECTION 2: The following questions ask about **symptoms and discomfort** that children may experience due to the **condition of their teeth, lips, mouth and jaws**.

ORAL SYMPTOMS

During the **last 3 months**, how often has your child had:

3. Pain in teeth, lips, jaws or mouth?
[ ] Never [ ] Once or twice [ ] Sometimes [ ] Often [ ] Everyday or almost everyday [ ] Don't know

5. Sores in the mouth?
[ ] Never [ ] Once or twice [ ] Sometimes [ ] Often [ ] Everyday or almost everyday [ ] Don't know

6. Bad breath?
[ ] Never [ ] Once or twice [ ] Sometimes [ ] Often [ ] Everyday or almost everyday [ ] Don't know

8. Food caught in or between the teeth?
[ ] Never [ ] Once or twice [ ] Sometimes [ ] Often [ ] Everyday or almost everyday [ ] Don't know

FUNCTIONAL LIMITATIONS

9. Difficulty biting or chewing foods such as fresh Apple, corn on the cob or firm meat?
[ ] Never [ ] Once or twice [ ] Sometimes [ ] Often [ ] Everyday or almost everyday [ ] Don’t know

10. Breathed through the mouth?
[ ] Never [ ] Once or twice [ ] Sometimes [ ] Often [ ] Everyday or almost everyday [ ] Don’t know

11. Had trouble sleeping?
[ ] Never [ ] Once or twice [ ] Sometimes [ ] Often [ ] Everyday or almost everyday [ ] Don’t know

14. Had difficulty drinking or eating hot or cold foods?
[ ] Never [ ] Once or twice [ ] Sometimes [ ] Often [ ] Everyday or almost everyday [ ] Don’t know

SECTION 3: The following questions ask about the effects that the **condition of children’s teeth, lips, mouth and jaws** may have on their **feelings** and **everyday activities.**

EMOTIONAL WELL-BEING

During the **last 3 months**, because of his/her **teeth, lips, mouth or jaws**, how often has your child been:

18. Irritable or frustrated?
[ ] Never [ ] Once or twice [ ] Sometimes [ ] Often [ ] Everyday or almost everyday [ ] Don’t know

19. Anxious or fearful?
[ ] Never [ ] Once or twice [ ] Sometimes [ ] Often [ ] Everyday or almost everyday [ ] Don’t know

28. Acted shy or embarrassed?
[ ] Never [ ] Once or twice [ ] Sometimes [ ] Often [ ] Everyday or almost everyday [ ] Don’t know

34. Concerned what other people think about his/her teeth, lips, mouth or jaws?
[ ] Never [ ] Once or twice [ ] Sometimes [ ] Often [ ] Everyday or almost everyday [ ] Don’t know

SOCIAL WELL-BEING

During the **last 3 months**, because of his/her **teeth, lips, mouth or jaws**, how often has your child been:

20. Missed school (e.g., pain, appointments, surgery)?
[ ] Never [ ] Once or twice [ ] Sometimes [ ] Often [ ] Everyday or almost everyday [ ] Don’t know

21. Had a hard time paying attention in school?
[ ] Never [ ] Once or twice [ ] Sometimes [ ] Often [ ] Everyday or almost everyday [ ] Don’t know

24. Avoided smiling or laughing when around other children?
[ ] Never [ ] Once or twice [ ] Sometimes [ ] Often [ ] Everyday or almost everyday [ ] Don’t know

29. Been teased or called names by other children?
[ ] Never [ ] Once or twice [ ] Sometimes [ ] Often [ ] Everyday or almost everyday [ ] Don’t know

SECTION 4: The following questions ask about effects that **a child's oral condition** may have on **PARENTS AND OTHER FAMILY MEMBERS**

During the **last 3 months**, because of your child's **teeth, lips, mouth or jaws**, how often have you or another family member:

36. Been upset?
[ ] Never [ ] Once or twice [ ] Sometimes [ ] Often [ ] Everyday or almost everyday [ ] Don't know

37. Had sleep disrupted?
[ ] Never [ ] Once or twice [ ] Sometimes [ ] Often [ ] Everyday or almost everyday [ ] Don't know

40. Had less time for yourself or the family?
[ ] Never [ ] Once or twice [ ] Sometimes [ ] Often [ ] Everyday or almost everyday [ ] Don't know

43. Been jealous of you or others in family?
[ ] Never [ ] Once or twice [ ] Sometimes [ ] Often [ ] Everyday or almost everyday [ ] Don't know

---

The numbering of the questions corresponds to the numbering of the original questionnaire [14–16].

Supplementary References

Jokovic, A.; Locker, D.; Stephens, M.; Kenny, D.; Tompson, B.; Guyatt, G. Measuring parental perceptions of child oral health-related quality of life. *J Public Health Dent*. **2003**, 63, 67-72. https://doi:10.1111/j.1752-7325.2003.tb03477.x.

Locker, D.; Jokovic, A.; Stephens, M.; Kenny, D.; Tompson, B.; Guyatt, G. Family impact of child oral and oro-facial conditions. *Community Dent Oral Epidemiol*. **2002,** 30, 438-48. doi:10.1034/j.1600-0528.2002.00015.x.
